# Acrylonitrile Butadiene Styrene (ABS) plastic‐based low cost tissue equivalent phantom for verification dosimetry in IMRT

**DOI:** 10.1120/jacmp.v11i1.3030

**Published:** 2009-12-17

**Authors:** Rajesh Kumar, S.D. Sharma, Sudesh Despande, Yogesh Ghadi, V.S. Shaiju, H.I. Amols, Y. S. Mayya

**Affiliations:** ^1^ Radiological Physics and Advisory Division Bhabha Atomic Research Centre CT&CRS Anushaktinagar Mumbai India; ^2^ P.D.Hinduja National Hospital and Research Centre Mumbai India; ^3^ Memorial Sloan‐Kettering Cancer Center New York USA

**Keywords:** IMRT, phantom, dose verification, ABS

## Abstract

A novel IMRT phantom was designed and fabricated using Acrylonitrile Butadiene Styrene (ABS) plastic. Physical properties of ABS plastic related to radiation interaction and dosimetry were compared with commonly available phantom materials for dose measurements in radiotherapy. The ABS IMRT phantom has provisions to hold various types of detectors such as ion chambers, radiographic/radiochromic films, TLDs, MOSFETs, and gel dosimeters. The measurements related to pretreatment dose verification in IMRT of carcinoma prostate were carried out using ABS and Scanditronix‐Wellhofer RW3 IMRT phantoms for five different cases. Point dose data were acquired using ionization chamber and TLD discs, while Gafchromic EBT and radiographic EDR2 films were used for generating 2D dose distributions. Treatment planning system (TPS) calculated and measured doses in ABS plastic and RW3 IMRT phantom were in agreement within ± 2%. The dose values at a point in a given patient acquired using ABS and RW3 phantoms were found comparable within 1%. Fluence maps and dose distributions of these patients generated by TPS and measured in ABS IMRT phantom were also found comparable both numerically and spatially. This study indicates that ABS plastic IMRT phantom is a tissue‐equivalent phantom and, dosimetrically, it is similar to solid/plastic water IMRT phantoms. Although this material is demonstrated for IMRT dose verification, it can also be used as a tissue‐equivalent phantom material for other dosimetry purposes in radiotherapy.

PACS number: 87.53Kn, 87.55Qr, 87.53Bn and 87.55Km

## I. INTRODUCTION

ICRU recommends that the dose delivery in radiotherapy should be accurate within ± 5%.^(1)^ IMRT is an advanced high‐precision radiotherapy technique which is used mainly for curative treatments. The principle of IMRT is to treat a patient from a number of different directions (or continuous arcs) with beams of nonuniform fluence, which have been optimized to deliver a high dose to the target volume and acceptably low dose to the surrounding normal structures.^(^ ^2^ ^–^ ^6^ ^)^ MLCs are commonly used for delivery of prescribed IMRT treatment either in a static or in a dynamic manner, which further complicates the procedure. Due to complex nature of IMRT treatment technique, pretreatment dose verification is an important aspect of IMRT QA program.^(^ ^7^ ^–^ ^12^ ^)^ An IMRT field often includes many small, irregular, off‐axis fields, requiring that a suitable IMRT phantom be used for dosimetric verification.^(^ ^13^ ^–^ ^14^ ^)^ Pretreatment verification of IMRT fields, therefore, requires the use of a dedicated tissue‐equivalent IMRT phantom with the provision of holding different types of detectors such as ionization chambers, TLDs, radiographic/radiochromic films, gel dosimeters, MOSFETs, and semiconductor diodes.

A number of IMRT phantoms with the facility for holding different types of detectors are available commercially for this purpose.^(^ ^15^ ^–^ ^18^ ^)^ The majority of these phantoms are made up of solid/plastic water material. Though these phantoms are suitable for pretreatment dose verification in IMRT, they are very costly and some have limited measurement options. Therefore there is a need to design and fabricate an IMRT phantom which is made up of tissue‐equivalent material, with options to verify the dose at a point and obtain dose distribution in 2D and 3D. In addition, the phantom should be made available at a reasonable price. In light of these requirements, a versatile IMRT phantom was designed and fabricated from a low cost tissue‐equivalent material. The tissue equivalency of a material for the dosimetry purpose depends on type and energy of the radiation. In the case of a photon beam, total attenuation coefficient; in the case of an electron beam, stopping power of the material should be comparable with tissue at the given energy of radiation.^(19)^ Acrylonitrile Butadiene Styrene (ABS) plastic was used as tissue‐equivalent material for fabrication of the IMRT phantom. This ABS plastic phantom was used for the pretreatment dose verification measurement in IMRT using ionization chamber, TLDs, radiochromic and radiographic film to demonstrate its suitability. A few measurements were also carried out with a commercially available Scanditronix‐Wellhofer IMRT RW3 phantom (IBA Dosimetry, Uppsala, Sweden) to compare the results obtained using an ABS IMRT phantom. This paper describes the design features of an ABS plastic IMRT phantom and the measurement results of pretreatment dose verification.

## II. MATERIALS AND METHODS

**Figure 1:**
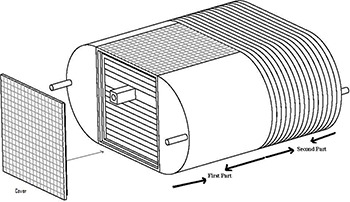
Schematic line diagram of ABS plastic IMRT phantom.

An IMRT phantom was designed and fabricated using tissue‐equivalent material commercially known as Acrylonitrile Butadiene Styrene (ABS) plastic. Cost of a sheet of 1.2 by 2.4 m^2^ of thickness 5 mm is about $200. ABS $\left[(C_{8}H_{8}•C_{4}H_{6}•C_{3}H_{3}N){\;}_{n}\right]$ is a copolymer made by polymerizing acrylonitrile and styrene in the presence of polybutadiene. The composition of ABS is: acrylonitrile −15% to 35%; butadiene – 5% to 30%; and styrene – 40% to 60%. Electron density of the phantom material relative to water was estimated by importing its CT scanned images to a recently calibrated treatment planning system for electron density, as well as by numerical evaluation using chemical composition of ABS. The total attenuation coefficient for the phantom material was also calculated using attenuation coefficient of its constituent elements. Table 1 presents dosimetry related physical parameters of commonly used phantom materials along with ABS for C60o gamma rays, and 6 and 15 MV X rays. These data indicate that physical parameters of the ABS are comparable to other standard phantom materials. The ABS phantom is elliptical in shape with dimensions sufficient to provide full scatter conditions similar to the irradiation of a patient. Figure 1 shows the schematic line diagram of ABS plastic IMRT phantom. This phantom has two parts. The first part has provision to incorporate ionization chambers, TLD discs, radiochromic films, and gel dosimeter; the second part has the provision to hold radiographic film only. The first part is designed in such a way that a cylindrical ionization chamber can be positioned within a 1 by 1 by 1 cm^3^ grid in the central region of the phantom. The positioning of the chamber within this grid is possible by moving suitable compensating inserts. Three fiducial marks are also available in the first part of the phantom for its reproducible positioning. The second part of the phantom consists of elliptical slices of 1 cm thickness and each of these slices contains three fiducial marks to identify the film orientation. Figure 2(a) shows the cubical insert of size 15 by 15 by 15 cm^3^ which has a cylindrical hole of 8 cm where the gel dosimeter container can be positioned. A large polymer gel sample can be used for the acquisition of dose distributions for entire target volumes. The large gel volume allows a dose distribution measurement of a large target volume and sometimes neighboring critical structures as well. Figure 2(b) shows the line diagram and Figure 2(c) shows the photograph of radiochromic film holder where a radiochromic film sample of 15 by 15 cm^2^ can be sandwiched between the plates to form a stack. This stack of radiochromic film can be used for the two‐dimensional as well as three‐dimensional dose distribution analysis. The ABS phantom has provision to hold a TLD tray in which TLDs of 4.5 mm in diameter and a thickness of 0.8 mm can be arranged at a spatial resolution of 7 mm (center to center). Figure 3 shows the final assembly of the fabricated ABS plastic IMRT phantom.

**Table 1:** Physical properties of common phantom materials and ABS plastic. The data for ABS plastic was numerically calculated from its elemental composition.

*Energy*	*Material*	*Effective Z*	*Electron Density Relative to Water*	*Physical Density*	*Attenuation Coefficient* $(\mu /p)\;(\times 10^{-2}\;{\text{cm}}^{2}/g)$
Co‐60	Water	7.420	1.000	1.00	6.323
	PMMA	6.467	1.034	1.19	6.143
	Polystyrene	5.690	0.969	1.06	6.123
	Solid water (WT1)	7.54	0.973	1.02	6.230
	RW‐3	–	0.967	1.04	6.110
	ABS	5.759	0.980	1.04	6.140
6MV	Water	7.420	1.000	1.00	4.940
	PMMA	6.467	1.034	1.19	4.796
	Polystyrene	5.690	0.969	1.06	4.780
	Solid water (WT1)	7.54	0.973	1.02	4.800
	RW‐3	–	0.967	1.04	4.767
	ABS	5.759	0.980	1.04	4.793
15MV	Water	7.420	1.000	1.00	3.030
	PMMA	6.467	1.034	1.19	2.919
	Polystyrene	5.690	0.969	1.06	2.890
	Solid water (WT1)	7.54	0.973	1.02	2.920
	RW‐3	–	0.967	1.04	2.889
	ABS	5.759	0.980	1.04	2.900

**Figure 2(a):**
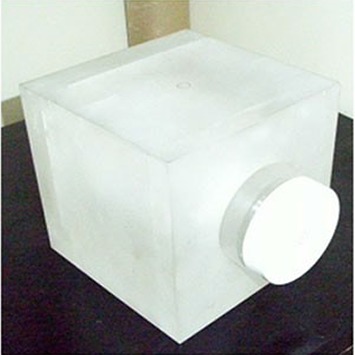
Holder for gel dosimeter container.

**Figure 2(b):**
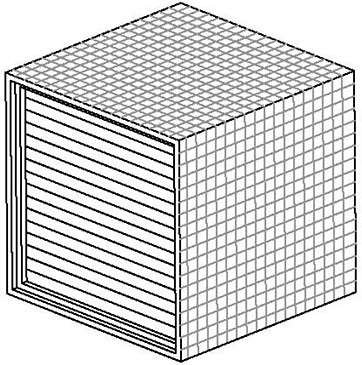
Schematic line diagram of radiochromic film holder.

**Figure 2(c):**
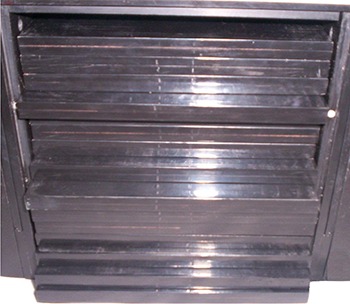
(c). Photograph of radiochromic film holder.

**Figure 3:**
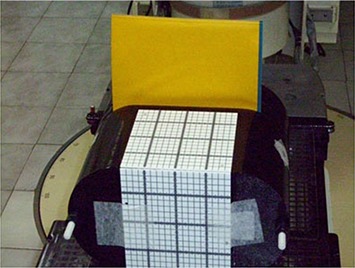
Final assembly of the locally fabricated IMRT phantom.

The suitability of ABS plastic phantom in IMRT dose verification was tested in comparison with a Scanditronix‐Wellhofer IMRT RW3 phantom. For this purpose, pretreatment dose verification was carried out for five different cases of carcinoma prostate treated by IMRT.

Volumetric CT scan (GE Discovery, WI, USA) of the ABS plastic phantom, with ionization chamber (FC65G; IBA Dosimetry, Uppsala, Sweden) located at 5 cm from its proximal surface, using the slice width of 3 mm was taken for the point dose verification. The volumetric CT scan data of the ABS plastic phantom was transferred to the Brain SCAN v5.2 (BrainLAB AG, Germany) treatment planning system. IMRT treatment plans of the patient of carcinoma prostate were planned on the phantom assuming the center of ion chamber as the centre of the tumor. The optimized plan so generated was transferred to the Varis Vision network system (Varian Medical Systems, Palo Alto, CA) and planned doses were delivered to the phantom at the predefined position of the chamber with 6 MV photon beam using Varian Clinac 2300 CD (Varian Medical Systems, Palo Alto, CA). The reading of the ionization chamber was converted into absorbed dose to water using methodology described in the IAEA TRS 398.^(20)^ Treatment plan and dose verification were carried out for five different cases of carcinoma prostate. The point of measurement was located in a low dose gradient zone for each of evaluated IMRT treatment plans. The experiment was repeated on a commercial IMRT phantom to compare the dose measurement carried out using the ABS plastic phantom.

A number of IMRT treatment plans were transferred to ABS plastic and Scanditronix‐Wellhofer RW3 IMRT phantom and doses at equal physical depth were calculated and measured to verify the equivalent of ABS plastic with RW3 as phantom material for dosimetry purpose.

To demonstrate the use of various detectors in the ABS IMRT phantom, the dose verification measurements were carried out using radiographic film, radiochromic film, and TLD along with ionization chamber. Radiographic film (EDR2 film, Eastman Kodak Company, Rochester, NY) was placed in the second part of the phantom and was irradiated as required by the planned IMRT treatment. Readout of the EDR2 film was carried out using VIDAR Dosimetry Pro Advantage scanner (VIDAR Systems Corporation, Herndon, VA). This way the EDR2 film gave the dose distribution data in the transverse plane. The calibration of EDR2 was carried out in 6 MV X‐ray beam. A 15 by 15 cm^2^ Gafchromic EBT film (Gafchromic EBT; ISP Inc. NJ, USA) was positioned in the first part of the phantom at a depth of 5 cm and was irradiated as required by the planned IMRT treatment. The irradiated film was read by flatbed scanner (EPSON Expression 10000XL; EPSON, UK). Scanning was done using the EPSON SCAN software with all filters switched off. The images were scanned in transmission mode and saved in RGB uncompressed tagged image file format (TIFF). For EBT films, the absorption peak falls in the red region and therefore the red component of the image was extracted to maximize film readout using ImageJ software (ImageJ 1.41o; National Institute of Health, USA). This way the Gafchromic EBT film gave the dose distribution data in the coronal plane. Two‐dimentional dosimetric analysis of scanned images was carried out using the IMRT dose verification software (OmniPro‐I'mRT version 1.5; IBA dosimetry, Uppsala, Sweden). To obtain a calibration curve for EBT film, the film samples of 4 by 4 cm^2^ were positioned in a conventional solid phantom perpendicular to the radiation beam and irradiated with 6 MV X rays in the known dose ranges: 50, 100, 200, 300 and 400 cGy. The exposed film samples were scanned in similar manner as mentioned above. A dose response curve was plotted and best fit of these data was used to determine unknown dose values from the knowledge of OD of exposed films using the polynomial expression: D=a*OD+b*OD^c where a, b and c are free fitting parameters.^(21)^

A TLD tray holding LiF: Mg, Ti (MTS‐N; TLD, Poland) discs 4.5 mm in diameter and with a thickness of 0.8 mm was position in the first part of the phantom at a depth of 5 cm and was irradiated as required by the planned IMRT treatment. The readouts of the TLD discs were taken 24 h after irradiation using a TLD reader (Rexon UL320, USA) with programmable temperature profile. The TL readout was taken in integration mode. The standard annealing and analysis process for the TLDs were followed to obtain dose data from the exposed TLDs. The calibration of TLD discs was also carried out in 6 MV X rays.

## III. RESULTS & DISCUSSION

Table 2 shows the TPS calculated dose values at a point in the Scanditronix‐Wellhofer IMRT RW3 phantom and the ABS plastic phantom for five different cases of prostate cancer treated by IMRT using 6 MV X rays. The table also includes ionization chamber measured dose values in these two phantoms at the same point where the dose was calculated by the TPS. A survey of data in this Table indicates that TPS calculated and ionization chamber measured dose values at corresponding points in Scanditronix‐Wellhofer IMRT RW3 phantom and ABS plastic phantom agree within 2% for all the five cases. If we assume TPS calculated dose as reference dose values, then it can easily be concluded from these observations that these two phantoms are quite suitable for pretreatment dose verification in IMRT.

**Table 2:** TPS calculated and ionization chamber measured dose values in Scanditronix‐Wellhofer RW3 and ABS plastic IMRT phantoms.

	*Calculated and ionization chamber measured dose values and their deviations*					
	*Scanditronix‐Wellhofer RW3 IMRT Phantom*			*ABS Plastic IMRT Phantom*		
*Case*	*TPS (Gy)*	*Ionization Chamber (Gy)*	*% Variation*	*TPS (Gy)*	*Measured Dose*	*% Variation*
1	2.28	2.32	−1.8	2.34	2.30	1.7
2	2.23	2.24	−0.45	2.22	2.26	1.8
3	2.1	2.06	1.9	2.11	2.07	1.9
4	2.23	2.22	0.45	2.19	2.20	−0.5
5	2.23	2.20	1.3	2.24	2.20	1.8

Table 3 lists the TPS calculated and ionization chamber measured dose values at 5 cm depth in Scanditronix‐Wellhofer IMRT RW3 phantom and ABS plastic phantom for five different cases of prostate cancer treated by IMRT using 6 MV X rays. The TPS calculated dose values in the two phantoms agree with each other within 0.5% for three cases, and shows a variation of 2.63% in one case. The ionization chamber measured dose values in these two phantoms agree within 1%. It can be concluded from the data in Table 3 that measured as well as calculated doses at a given point in these two phantoms are in agreement with each other. As Scanditronix‐Wellhofer IMRT RW3 phantom is a tissue‐equivalent phantom; the ABS plastic IMRT phantom can also be assumed to be a tissue‐equivalent phantom. However, the cost of ABS plastic phantom is about 8 to 10 times lower than the cost of the Scanditronix‐Wellhofer IMRT RW3 phantom and similar other commercial phantoms with equivalent features.

**Table 3:** TPS calculated and ionization chamber measured dose values at 5 cm depth in Scanditronix‐Wellhofer RW3 and ABS plastic IMRT phantoms.

	*Calculated and measured dose and their deviations*					
	*TPS (Gy)*			*Ionization Chamber (Gy)*		
*Case*	*Scanditronix‐Wellhofer RW3 Phantom*	*ABS plastic IMRT Phantom*	*% Deviations*	*Scanditronix‐Wellhofer RW3 Phantom*	*ABS Plastic IMRT Phantom*	*% Deviations*
1	2.28	2.34	−2.63	2.32	2.30	0.86
2	2.23	2.22	0.45	2.24	2.26	−0.89
3	2.1	2.11	−0.48	2.06	2.07	−0.49
4	2.23	2.19	1.79	2.22	2.2	0.90
5	2.23	2.24	−0.45	2.2	2.2	0

**Figure 4:**
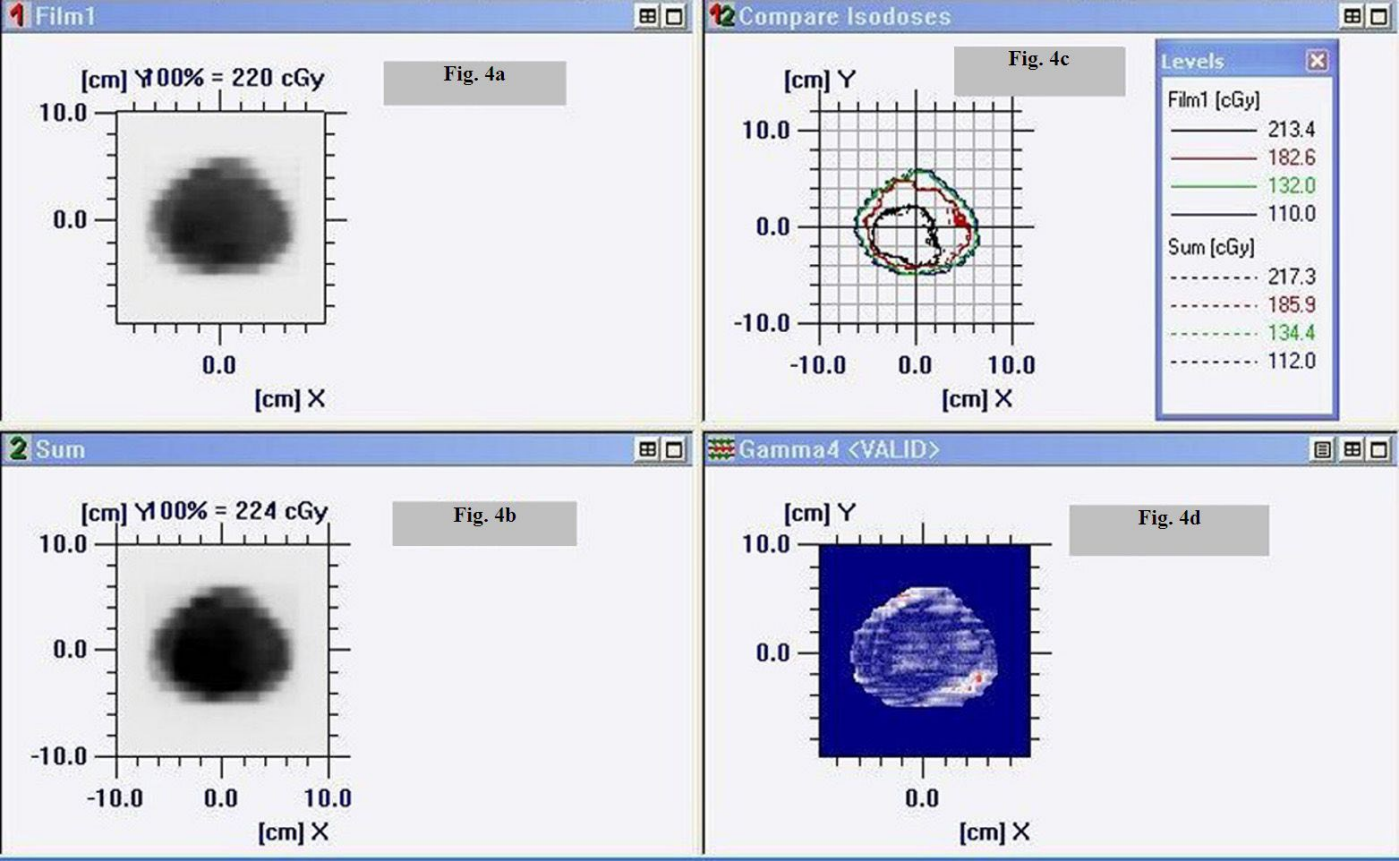
Graphic showing: (a) fluence map in coronal plane recorded on Gafchromic EBT film of an IMRT plan in ABS plastic phantom; (b) TPS‐generated fluence map in coronal plane of an IMRT plan; (c) comparison of isodose lines recorded on Gafchromic EBT film in ABS plastic phantom and TPS‐generated isodose line of an IMRT plan in coronal plane; and (d) gamma analysis of an IMRT plan.

**Figure 5:**
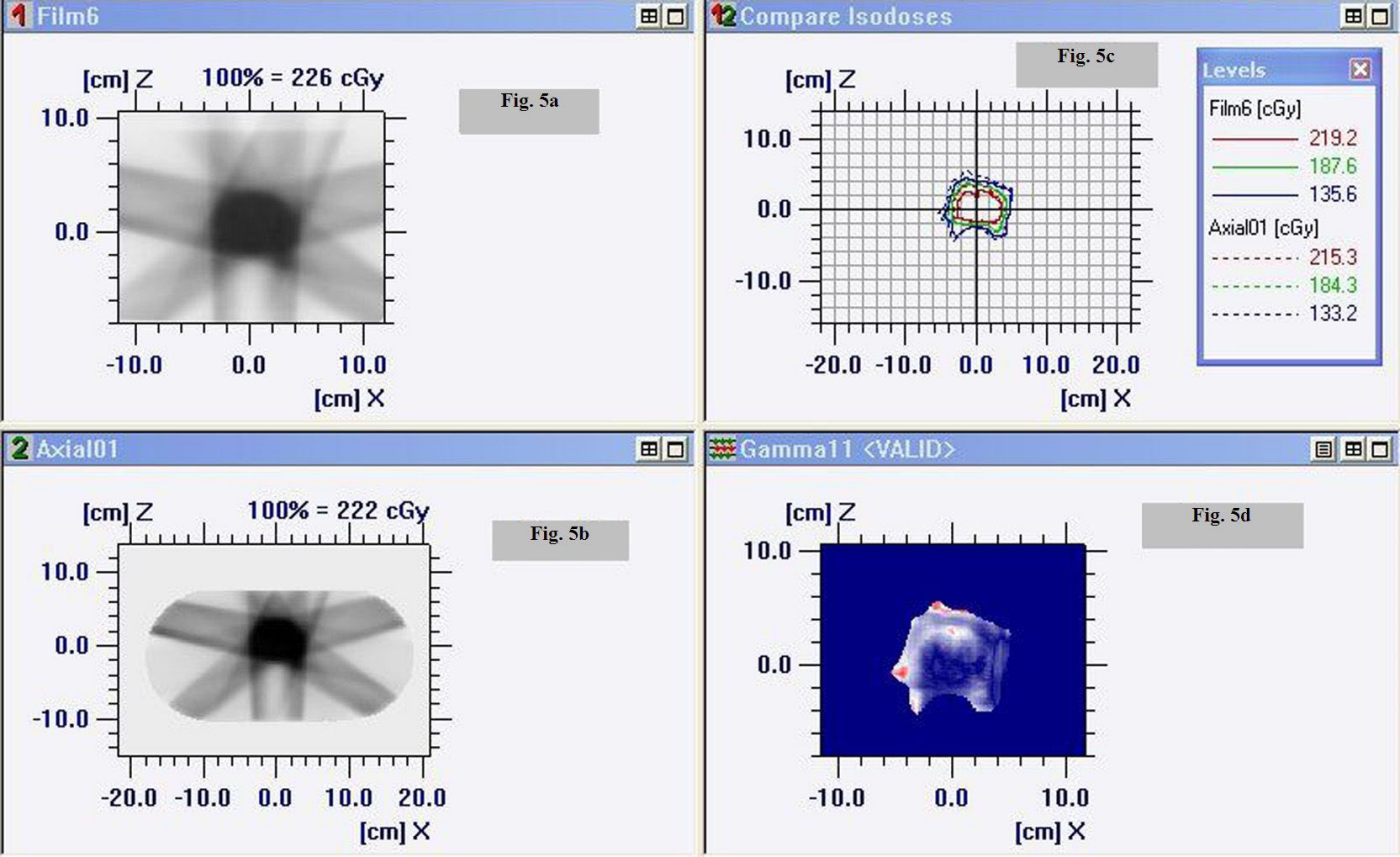
Graphic showing: (a) fluence map in transverse plane recorded on EDR2 film of an IMRT plan in ABS plastic phantom; (b) TPS‐generated fluence map in coronal plane of an IMRT plan; (c) comparison of isodose lines recorded on EDR2 film in ABS plastic phantom and TPS‐generated isodose line of an IMRT plan in transverse plane; and (d) gamma analysis of an IMRT plan.

Point doses measured using EBT Gafchromic film in ABS plastic IMRT phantom were found to be within 0.6% in comparisons of TPS calculated dose value. However, the dose measured using TLD discs at the same point were found to be within 2.8%. Figure 4 shows: (a) fluence map in coronal plane recorded on Gafchromic EBT film of an IMRT plan in ABS plastic phantom; (b) TPS‐generated fluence map in coronal plane of an IMRT plan; (c) comparison of isodose lines recorded on Gafchromic EBT film in ABS plastic phantom and TPS‐generated isodose line of an IMRT plan in coronal plane; and (d) gamma analysis of an IMRT plan. Figure 5 shows: (a) fluence map in transverse plane recorded on EDR2 film of an IMRT plan in ABS plastic phantom; (b) TPS‐generated fluence map in coronal plane of an IMRT plan; (c) comparison of isodose lines recorded on EDR2 film in ABS plastic phantom and TPS‐generated isodose line of an IMRT plan in transverse plane; and (d) gamma analysis of an IMRT plan. The correlation coefficient of plans recorded on Gafchromic EBT/ EDR 2 films in ABS plastic phantom were found to be better than 0.990 for the region of interest. The measured dose distribution by

EBT/ EDR2 films agreed in the region of interest with a planned dose distribution that passed 3%, 3 mm gamma‐index evaluation. Figures 4 and 5 indicate close agreement between the information recorded on Gafchromic EBT/ EDR2 films in ABS plastic phantom and TPS‐generated data for the IMRT plan of a patient.

## IV. CONCLUSIONS

A novel IMRT phantom was designed and fabricated using ABS plastic. The studies carried out on this phantom indicate that it is equivalent to plastic/solid water IMRT phantoms available commercially. The ABS phantom is a versatile tissue‐equivalent phantom which can be used for pretreatment dose verification in IMRT using different types of radiation detectors. The phantom is suitable for dosimetry in 1D, 2D and 3D. Though this material is demonstrated for IMRT dose verification, it can also be used as a tissue‐equivalent phantom material for other dosimetry purposes in radiotherapy.

## ACKNOWLEDGEMENT

This work is partially supported by the International Atomic Energy Agency (IAEA).
